# Assessing directional connections between symptoms, cognition, insight, and real-life functioning in schizophrenia: a partial ancestor graphs analysis

**DOI:** 10.3389/fpsyt.2026.1782281

**Published:** 2026-05-13

**Authors:** Claudio Brasso, Gianluca Colli, Silvio Bellino, Paola Bozzatello, Cristiana Montemagni, Paola Rocca

**Affiliations:** 1 Department of Neuroscience ‘Rita Levi Montalcini’, University of Turin, Turin, Italy; 2 Struttura Complessa (S.C.) Psichiatria U, Dipartimento di Neuroscienze e Salute Mentale, Azienda Ospedaliero-Universitaria (A.O.U.) Città della Salute e della Scienza di Torino, Turin, Italy

**Keywords:** association, causality, directionality, learning, metacognition, negative symptoms, working memory

## Abstract

**Introduction:**

Schizophrenia is a severe chronic mental disorder causing significant global disability. Understanding the intricate relationships between symptoms, cognitive functions, and real-life outcomes is essential for developing effective interventions. Prior research, while informative, could not often determine the direction of the association between these illness-related factors. This study aimed to investigate the possible causal connections within the interrelationships of these variables.

**Methods:**

This cross-sectional study included 215 clinically stable patients diagnosed with schizophrenia. Comprehensive assessments covered psychopathology, neurocognition, social cognition, metacognition, clinical insight, and real-life functioning. Causal relationships were explored using Partial Ancestral Graphs, a causal discovery framework that accounts for mediators and confounders. The Greedy Fast Causal Inference algorithm was employed with 1,000 bootstrap replications to assign edge orientations.

**Results:**

A central neurocognitive–metacognitive–functional system of directed connection emerged: visual learning was linked to attention/vigilance and working memory. Working memory showed a direct relationship with metacognition, which, in turn, was connected to real-life functioning. Two partly independent contributions to real-life functioning were also identified: conceptual disorganization and experiential negative symptoms, which were directly related to expressive deficits. Positive symptoms, depressive symptoms, and social cognition occupied peripheral positions, showing no significant connection with other variables. Unawareness and misattribution of symptoms showed an indeterminate association disconnected from the main network.

**Discussion:**

The findings show a set of directed associations that start with neurocognitive abilities, pass through working memory and metacognition, and terminate in real-life functioning. Independently, conceptual disorganization and expressive negative symptoms also exert direct influences. These directed systems of connections provide operational guidance for clinical practice, highlighting critical targets for interventions such as cognitive remediation focused on working memory, metacognitive therapies, and strategies addressing disorganization and avolition, all aimed at improving real-life outcomes in schizophrenia.

## 1 Introduction

Schizophrenia is a chronic and complex mental disorder that consistently ranks among the leading causes of disability worldwide, contributing substantially to the global burden of disease (1–3), involving significant consequences for psychosocial functioning, relationships, employment, and the management of everyday activities (4, 5). In recent years, real-life functioning has been increasingly recognized as one of the most relevant clinical outcomes, as a crucial indicator of recovery (6, 7). However, only about 57% of individuals with a first-episode psychosis (FEP) and about 38% of subjects with a history of multiple illness episodes achieve good functioning, despite symptom reduction (8), highlighting the need to investigate the relationships among the different domains involved in the disorder to clarify the mechanisms underlying real-life functioning.

The most consistent predictors of functional impairment among the symptom domains are represented by negative symptoms in their motivational and expressive components (9, 10) and by disorganized, depressive, and anxiety symptoms (11). Neurocognition shows a strong association with negative symptoms and a weaker one with positive symptoms (12–14). Disorganization is also associated with cognitive difficulties and poorer performance on Theory of Mind and emotion recognition tasks (15–17). Lower levels of metacognitive capacity are associated with greater symptom severity (18, 19). Longitudinal studies have also shown that metacognitive deficits can predict symptom progression, with particularly consistent signs for negative symptoms (20, 21). Studies investigating the relationship between cognition and functioning have highlighted neurocognition (22–26) as an important determinant of the variability in real-life behavior. This role might be supported (24), interconnected (27, 28), or even mediated (25) by social cognition, which allows cognitive abilities to be translated into the context of everyday life, and which alone represents another determinant factor for functioning (4, 25, 28, 29). Metacognition is a relevant predictor of functioning (28, 30, 31) and a key mediator of the relationship between neurocognition and functional outcomes as well (25, 32), indicating that the ability to integrate and reflect on one’s own mental and cognitive processes can in fact mediate the impact of these abilities on real-life outcomes. The decisive role of these domains in functioning is particularly relevant in schizophrenia, where cognitive deficits are present in the majority of patients, often emerging before or during the first episode of psychosis and remaining relatively stable throughout the course of the illness (33, 34), and where stable impairments are also found in social cognition (35–38) and in specific metacognitive abilities (39–41). These interactions between domains were initially explored using correlational approaches and subsequently using structural equation modeling (SEM), which allows simultaneous evaluation of direct and indirect effects and hypothesized mediation chains among cognitive domains, symptoms, and functioning. Brekke et al. (42) proposed a “biosocial” model, positing that neurocognition, social cognition, social competence, and environmental support interact to predict real-life functioning in the short- and medium-term. Many authors clarified the relevance of many domains, such as functional capacity (23), neurocognition, and social cognition (4, 24, 43, 44) in this pathway (45, 46). The limitations of structural equation modeling (SEM), particularly its reliance on *a priori* hypotheses about relationships between variables, which may obscure unexamined or more complex connections, have led to growing interest in network analysis, a data-driven and exploratory approach that represents complex systems without assuming predefined causal hierarchies, laying the groundwork for causal modeling.

Early psychiatric applications were mainly symptom-focused, identifying interconnected constellations with “central” or bridging symptoms (47). Multidomain network studies in schizophrenia demonstrated that social cognition, neurocognition, resilience, and real-life functioning represent distinct yet interconnected constructs, with functional capacity and everyday skills transmitting the effects of cognitive and symptomatic deficits to other domains. These configurations proved stable over time and were replicated in independent samples (6, 48, 49). Theoretical work further emphasized the ability of networks to capture the internal structure of psychopathological systems, overcoming the rigidity of predetermined models (31, 50–52).

Network analysis is limited by its inability to determine causal direction or distinguish direct from latent effects. Directed Acyclic Graphs (DAGs) formalize causal hypotheses through acyclic directed edges, explicitly modeling pathways, confounders, and biases (53), but they cannot account for latent-variable uncertainty. To overcome this, probabilistic graphical models oriented toward causality, particularly Partial Ancestral Graphs (PAGs), incorporate partially oriented relations and the possibility of unobserved confounding (54). Based on assumptions such as the causal Markov condition, faithfulness, and acyclicity, PAGs infer plausible causal pathways from observational data, typically cross-sectional, producing hypotheses that require longitudinal or experimental validation (55). Despite these limitations, the use of PAGs in psychiatry, still in its early stages, is promising. In a first-episode schizophrenia sample, Miley et al. (56) found that socio-affective capacity caused motivation, which in turn influenced social and occupational functioning, revealing causal chains difficult to anticipate *a priori*. A significant contribution by Giuliani et al. (57) applied a PAG-based causal discovery procedure to a large cohort of over six hundred patients with chronic schizophrenia, examining cognitive, social-cognitive, symptomatic, and functional domains. Working memory emerged as the primary causal node, exerting downstream effects on other cognitive domains and on functional capacity, which in turn mediated the association with everyday functioning and influenced interpersonal and work skills, as well as disorganization and negative symptoms. This configuration remained stable over a four-year period, suggesting that the identified pathways reflect stable illness-related features rather than cross-sectional clinical states.

Based on these premises, the Partial Ancestral Graph (PAG) approach is particularly suited to complex disorders such as schizophrenia, characterized by numerous, interrelated clinical and cognitive variables often influenced by unobservable or unstudied factors. Accordingly, this study aimed to investigate the directionality of relationships among symptoms, cognition, and functioning in schizophrenia using a causal discovery framework grounded in PAGs.

## 2 Methods

### 2.1 Participants

This cross-sectional study included 215 patients diagnosed with schizophrenia according to DSM-5 criteria (58). Participants were recruited from the Psychiatric University Unit “Struttura Complessa Psichiatria Universitaria, Dipartimento di Neuroscienze e Salute Mentale, Azienda Ospedaliero-Universitaria Città della Salute e della Scienza di Torino, Presidio Molinette”.

Inclusion criteria were: (1) schizophrenia diagnosis according to DSM-5 (58), confirmed by two senior clinicians (CB and CM) using the Structured Clinical Interview for DSM-5, Research Version (SCID-5-RV) (59); (2) age between 18 and 65 years; and (3) clinical stability, defined as no hospitalization or antipsychotic treatment changes in the three months preceding enrollment. Exclusion criteria included: (1) history of severe head trauma and (2) moderate-to-severe intellectual development disorder or neurological comorbidity.

All patients were receiving pharmacological treatment consistent with current APA guidelines (60). Written informed consent was obtained after a full explanation of the study. The study complied with the Declaration of Helsinki, ICH-GCP guidelines (61), and was approved by the local ethics committee (Protocol number: 0126325).

### 2.2 Assessment

Sociodemographic variables included sex, age, and years of education. Clinical variables comprised illness duration, number of hospitalizations, substance use, and ongoing pharmacological treatment (including atypical and long-acting injectable antipsychotics).

Positive and disorganized symptoms were evaluated with the Positive and Negative Syndrome Scale (PANSS) (62). Positive symptoms were derived according to the five-factor model proposed by Wallwork et al. (63), while disorganization was estimated with the PANSS item P2 ‘conceptual disorganization’ only to minimize overlap with cognitive performance (64).

Negative symptoms were assessed using the Italian validated version of the Brief Negative Symptom Scale (BNSS) (65, 66). Two principal dimensions of negative symptoms were rated: experiential negative symptoms, consisting of anhedonia, asociality, and avolition/apathy, and expressive negative symptoms, including blunted affect and alogia.

Depressive symptoms were measured using the Calgary Depression Scale for Schizophrenia (CDSS) (67); the total score was used in analyses.

Neurocognitive functioning was assessed with the MATRICS Consensus Cognitive Battery (MCCB) (68), which evaluates processing speed, attention/vigilance, working memory, verbal and visual learning, reasoning/problem-solving, and social cognition in terms of emotion management. Each specific domain score was used for analysis.

Metacognitive ability was evaluated with the Metacognition Assessment Scale (69, 70), based on transcripts of a narrative clinical interview. The MAS includes four domains: self-reflection, understanding others’ minds, decentration, and mastery. The total MAS score was analyzed.

Clinical insight was assessed with the Scale to Assess Unawareness of Mental Disorder (SUMD) (71), comprising three global and 17 symptom-specific items rated from full awareness to complete unawareness (71, 72). The scale includes seven dimensions: the three global, i.e., awareness of mental disorder, awareness of symptom consequences, awareness of treatment need, and four symptom-specific ones: present and past unawareness of symptoms (UoS), and present and past misattribution of symptoms (MoS). Only current UoS and MoS were analyzed, with scores averaged across present items (i.e., current symptoms) to reduce retrospective bias and capture the most informative insight dimensions (71, 72).

Real-life functioning was measured with the Italian version of the Specific Level of Functioning Scale (SLOF) (73, 74), an informant-rated tool with 43 items, organized into six domains: physical functioning, self-care, interpersonal relationships, social acceptability, everyday activities, and work skills. The total score was analyzed.

### 2.3 Statistical analysis

We applied Partial Ancestral Graphs (PAGs) (54, 55) to identify causal connections between symptoms, cognitive domains, and functioning. PAG edges represent ancestral relations among network nodes and carry multiple specific causal meanings, as graphically represented by the tail, the head, and the edge line. Each edge connects two nodes, i.e., variables, one in correspondence with the tail and one with the head. PAGs can take into account multiple aspects of these relationships, including the degree of certainty about the directionality of the connection and the presence of unmeasured third variables, namely, confounders and mediators. In a PAG, edge endpoints can take three forms, each encoding distinct causal information (54, 55): 1) Straight Tail (–) indicates that the corresponding variable is causally prior to the other; it may be an ancestor or, at minimum, not a descendant; 2) Arrow (>) indicates that the variable it points to cannot be an ancestor of the other, excluding reverse causation; 3) Circle (o) indicates uncertainty regarding orientation and its position is constrained but not fully determined. Arrows and circles could be present in the tail and/or in the head of an edge (55).

As an example, we present four types of directionality in the relationship (edge) between the variables (nodes) A and B:

- *A −> B* indicates that A is an ancestor of B, i.e., a causal influence of A on B is consistent with the data;
- *A o−> B* represents an uncertain orientation where B cannot causally precede A;
- *A o−o B* represents an undirected orientation;
- *A <−> B* represents an uncertain association.

Edge lines encode information about confounders and mediators. A solid line indicates that a confounder does not explain the association, while a dashed line indicates that a latent confounder may exist. A thick line represents a direct relation of A on B among observed variables. In contrast, a thin line indicates a possibly direct relationship, suggesting that other observed variables may mediate it.

Thus, PAGs compactly summarize both directionality and the quality of causal inference, including visibility relative to confounders and mediators. For clarity, the main edge configurations and their interpretative meanings are summarized in **Table 1**.

**Table 1:** Main configurations of associative edges identifiable in PAG analysis.

Type of edge N.	Edge configuration	Edge description	Meaning
1	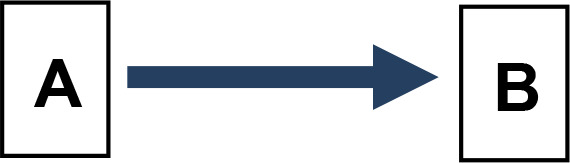	Definitely visible (solid line) Definitely direct (thick line)	- A is a direct cause of B AND - B is not the cause of A AND - There is no unmeasured confounder of A and B AND - There is no mediator between A and B.
2	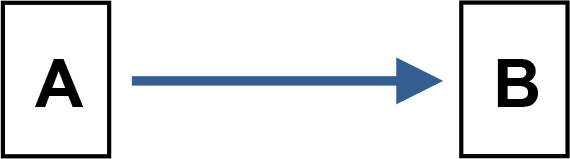	Definitely visible (solid line) Possibly direct (thin line)	- A is a direct or indirect (i.e., there might be a mediator) cause of B AND - B is not the cause of A AND - There is no unmeasured confounder of A and B.
3	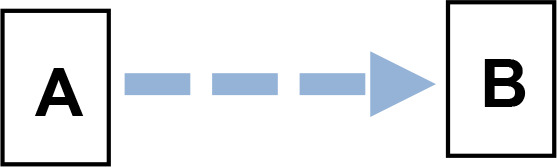	Not definitely visible (dashed line) Definitely direct (thick line)	- A is a direct cause of B AND - B is not the cause of A AND - An unmeasured confounder of A and B is possible.
4	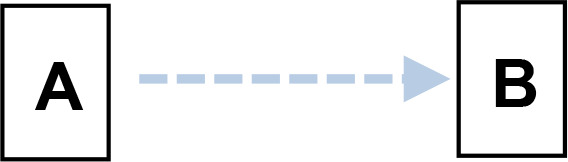	Not definitely visible (dashed line) Possibly direct (thin line)	- A is a direct or indirect (i.e., there might be a mediator) cause of B AND - B is not the cause of A AND - An unmeasured confounder of A and B is possible.
5	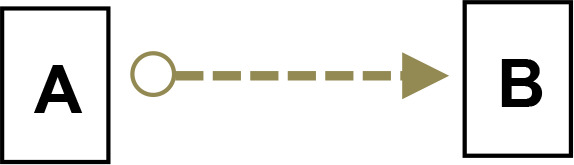	Uncertain orientation on the A side	- A is a cause of B, OR - There is an unmeasured confounder that causes both A and B, OR - Both conditions hold, AND - B is not the cause of A.
6	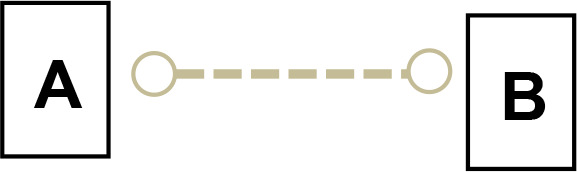	Uncertain orientation on both sides Not definitely direct, and visibility is not guaranteed.	One of the following holds: A is a cause of B, OR B is a cause of A, OR There is an unmeasured confounder that causes both A and B, OR Both (a) and (c) are true, OR: Both (b) and (c) are true.
7	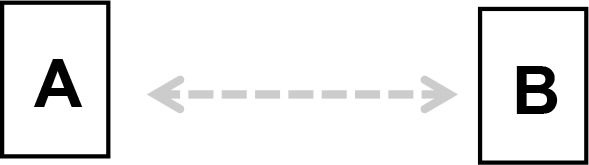	Double arrow	Undetermined relationship. There is an unmeasured confounder that causes the observed relationship between A and B.

A and B represent two variables. Blue tones indicate direct relationships between variables (Edges N. 1 and 2). The deepest blue indicates the absence of both confounders and mediators (Edge N. 1). The less deep blue indicates the possibility of a mediator (Edge N.2). The two lighter blues indicate the possible presence of confounders (Edges N. 3 and 4). The lightest blue indicates the possibility of both a confounder and a mediator (Edge N.4). Brown indicates an uncertain orientation for one variable (Edge N. 5); light brown for both (Edge N. 6). Gray indicates an undetermined relationship (Edge N.7).

To identify causal relationships, we employed the software Tetrad (version 7.6.0; https://sites.google.com/view/tetradcausal). We applied the Greedy Fast Causal Inference (GFCI) algorithm (75), which integrates the strengths of Fast Causal Inference (FCI) and Fast Greedy Equivalence Search (FGES). In the first phase, FGES constructs a preliminary “supergraph” by progressively adding edges to an empty graph through a score-based procedure to identify potential causal adjacencies. In the second phase, FCI refines this structure by removing spurious or indirect associations and highlighting potential latent confounding variables. The stability of the resulting structure was evaluated using bootstrap resampling with 1,000 replications. GFCI was applied to the resulting 1,000 subsamples, each containing 90% of the original data. For each edge, the probability of occurrence across replications was estimated; edges with probabilities greater than 0.5 were retained, with orientation assigned according to the most frequently observed edge type permitted by the PAG formalism in Tetrad. This approach allows assessment of the robustness and variability of causal structures identified by GFCI.

GFCI implementation was performed using both the scoring with the Bayesian Information Criterion (BIC) and conditional independence testing with Fisher’s Z test. In BIC-based estimation, the penalty discount parameter was set to 2 to account for the high dimensionality and noise of the observational data, reducing overfitting and discouraging the inclusion of spurious connections. Fisher’s Z test was applied with a significance threshold of 0.05 (57).

## 3 Results

### 3.1 Sample description

The sample consisted of 215 participants, including 69 females (32.1%). The mean age was 36.0 years (SD = 13.0), mean education 12.2 years (SD = 3.1), mean illness duration 11.2 years (SD = 11.8), and mean number of hospitalizations 3.5 (SD = 4.5). Clinically, 42 participants (19.5%) had a comorbid substance use disorder, 183 (85.1%) were receiving atypical antipsychotics, and 51 (23.7%) were treated with long-acting injectable (LAI) formulations.

Comprehensive data on clinical, cognitive, and functional characteristics are presented in **Table 2**.

**Table 2:** Sample characteristics.

Descriptive variables	Mean (SD)/ N (%)
Sociodemographic variables	
Female gender, n (%)	69 (32.1%)
Age, years, mean (SD)	36.00 (13.04)
Education, years, mean (SD)	12.23 (3.18)
Clinical variables	
Illness duration, years, mean (SD)	11.17 (11.79)
Number of hospitalizations, mean (SD)	3.45 (4.53)
Substance use disorder, n (%)	42 (19.5%)
Atypical antipsychotic treatment, n (%)	183 (85.1%)
Long-acting injectable antipsychotics, n (%)	51 (23.7%)
Symptoms	
Positive symptoms (PANSS factor), mean (SD)	9.43 (4.15)
Conceptual disorganization (PANSS item P2), mean (SD)	2.60 (1.48)
Avolition (BNSS factor), mean (SD)	19.46 (9.32)
Expressive deficits (BNSS factor), mean (SD)	13.54 (7.68)
Depression (CDSS total), mean (SD)	3.95 (4.60)
Cognition	
Processing speed (MCCB), percentile, mean (SD)	25.81 (8.3)
Working memory (MCCB), percentile, mean (SD)	32.82 (11.36)
Attention/vigilance (MCCB), percentile, mean (SD)	30.01 (11.02)
Reasoning and problem solving (MCCB), percentile, mean (SD)	34.42 (8.62)
Verbal learning (MCCB), percentile, mean (SD)	34.70 (9.61)
Visual learning (MCCB), percentile, mean (SD)	37.39 (14.52)
Emotion management (MSCEIT, MCCB), percentile, mean (SD)	32.79 (11.46)
Metacognition (MAS total), mean (SD)	15.20 (7.77)
Clinical insight	
Unawareness of current symptoms (SUMD), mean (SD)	2,91 (2,03)
Misattribution of current symptoms (SUMD), mean (SD)	3,44 (2,32)
Real-life functioning	
Real-life functioning (SLOF total score), mean (SD)	181.27 (20.77)

SD, standard deviation; N, absolute number; BNSS, Brief Negative Symptoms Scale; CDSS, Calgary Depression Scale for Schizophrenia; MAS, Metacognition Assessment Scale; MCCB, MATRICS Consensus Cognitive Battery; MSCEIT, Mayer-Salovey-Caruso Emotional Intelligence Test; PANSS, Positive and Negative Syndrome Scale; SD, Standard Deviation; SLOF, Specific Level Of Functioning scale; SUMD, Scale to Assess Unawareness of Mental Disorder.

### 3.2 Partial Ancestral Graphs

**Figure 1:**
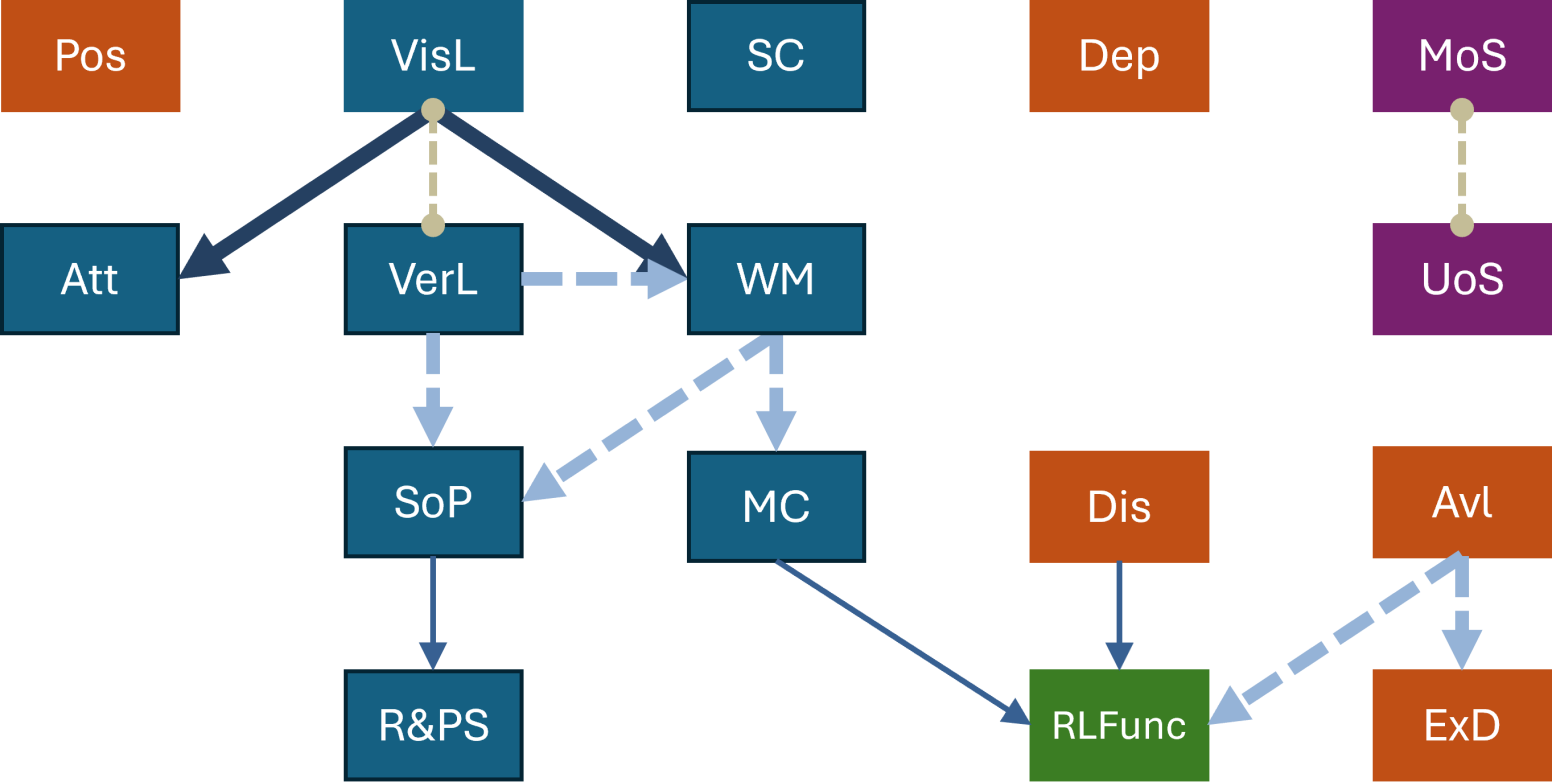
Causal inference: edge types in PAGs among included variables. tt, Attention/Vigilance; Avl, Avolition factor (i.e., experiential negative symptoms); Dis, Conceptual Disorganization; ExD, Reduced Emotional Expressivity (i.e., expressive negative symptoms); MC, Metacognition abilities; MoS, Misattribution of Symptoms; R&PS, Reasoning and Problem Solving; RLFunc, Real-Life Functioning; SoP, Processing Speed; UoS, Unawareness of Symptoms; VerL, Verbal Learning; VisL, Visual Learning; WM, Working Memory. Symptoms are represented in red, cognitive variables in blue, clinical insight in purple, and functioning in green.
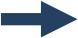
Edge type N.1: direct relationship from variable A to B without confounders and mediators;
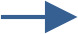
Edge type N.2: direct relationship from variable A to B without confounders, but with a possible mediator;
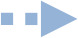
Edge type N.3: direct relationship from variable A to B with a possible confounder, but without mediators;
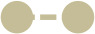
Edge type N.6: indirect relationship between variables A and B.

**Table 3** reports the bootstrap results of the Partial Ancestral Graph (PAG) analysis. For each pair of variables (nodes), the table shows the probability of an edge existence, as well as the distribution of edge types observed across 1,000 bootstrap replications. The most frequent edge configuration (i.e., the one most frequently detected during bootstrapping) was retained and used to construct the final graph shown in **Figure 1**.

**Table 3:** Frequency of the different types of edges detected.

Variable A	Most frequent type of interaction (edge type N. and direction ➔ or o-o from variable 1 to 2)	Variable B	Probability that the interaction is present	N.1 ➔ DD NLC	N.2 ➔ PD NLC	N.3 ➔ DD PLC	N.6 o-o
WM	**N.3 ➔ DD PLC**	MC	0.888	–	–	**0.563**	0.027
Dis	**N.2 ➔ PD NLC**	RLFunc	0.932	0.207	**0.438**	–	0.195
Avl	**N.3 ➔ DD PLC**	ExD	0.948	–	–	**0.430**	0.279
MC	**N.2 ➔ PD NLC**	RLFunc	0.689	–	**0.375**	–	0.062
MoS	**N.6 o-o**	UoS	0.960	–	0.233	–	**0.364**
Avl	**N.3 ➔ DD PLC**	RLFunc	0.698	–	–	**0.319**	0.125
SoP	**N.2 ➔ PD NLC**	R&PS	0.907	–	**0.313**	–	0.267
VerL	**N.3 ➔ DD PLC**	SoP	0.871	–	–	**0.305**	0.259
VisL	**N.1 ➔ DD NLC**	Att	0.697	**0.290**	–	–	0.099
VisL	**N.1 ➔ DD NLC**	WM	0.891	**0.268**	–	–	0.191
WM	**N.3 ➔ DD PLC**	SoP	0.805	–	–	**0.238**	0.215
VerL	**N.3 ➔ DD PLC**	WM	0.763	–	–	**0.234**	0.222
VisL	**N.6 = o-o**	VerL	0.825	–	0.167	–	**0.219**

Edge type N.1 = ➔ DD NLC: Probability of an edge from variable A to variable B, Definitely Direct (no mediator), No Latent Confounder (definitely visible); represented with a solid thick line with the darkest blue.

Edge type N.2 = ➔ PD NLC: Probability of an edge from variable A to variable B, Possibly Direct (possible mediator), No Latent Confounder (definitely visible); represented with a solid thin line with the less dark blue.

Edge type N.3 = ➔ DD PLC: Probability of an edge from variable A to variable B; Definitely Direct (no mediator); Possible Latent Confounder (not definitely visible); represented with a dashed thick line with the less light blue.

Edge type N.4 = o-o: Probability of indeterminate relation; direction unresolved. Represented with light brown.

Interaction types that have never been found to be the most common for any pair of variables are not reported. Bold numbers indicate the most frequent type of association in the bootstrapping; - indicates a probability < 0.0005.

Att, Attention/Vigilance; Avl, Avolition factor (i.e., experiential negative symptoms); Dis, Conceptual Disorganization; ExD, Reduced Emotional Expressivity (i.e., expressive negative symptoms); MC, Metacognition abilities; MoS, Misattribution of Symptoms; R&PS, Reasoning and Problem Solving; RLFunc, Real-Life Functioning; SoP, Processing Speed; UoS, Unawareness of Symptoms; VerL, Verbal Learning; VisL, Visual Learning; WM, Working Memory.

Symptoms are represented in red, cognitive variables in blue, clinical insight in purple, and functioning in green.

In this representation, variables are connected by edges oriented according to the bootstrap-derived most frequent direction (**Table 3**) using the graphical representation described in **Table 1**.

Certain domains—namely, positive symptoms (Pos), depressive symptoms (Dep), and social cognition (SC)—did not show significant connections with other nodes, suggesting that, within our sample, they do not play a direct role in organizing the relationships among symptoms, cognitive domains, and functioning.

Within the main network, visual learning (VisL) emerged as the first node in the group of connections among cognitive domains. It showed direct connections to attention/vigilance (Att) and working memory (WM), as well as an indeterminate relation (o–o edge) with verbal learning (VerL). The latter showed a direct connection—though potentially affected by latent confounding—with two other cognitive variables: processing speed (SoP), which in turn connected to reasoning, and problem solving (R&PS). In these associations, latent confounders could be excluded, although intermediate mediators are possible. Working memory was also linked to metacognition (MC), which, in turn, was connected to real-life functioning (RLFunc) in a potentially mediated, but not confounded, manner.

Conceptual disorganization (Dis) showed a directed association with real-life functioning (RLFunc), free from latent confounding but possibly mediated by third factors, indicating an independent contribution to reduced functioning. Avolition (Avl)—encompassing experiential negative symptoms, i.e., avolition, anhedonia, and asociality—also emerged as a key node, showing direct, though potentially confounded, connections with both real-life functioning (RLFunc) and expressive deficits (ExD).

Finally, an isolated association was observed: misattribution of symptoms (MoS) correlated with unawareness of symptoms (UoS), although the relation was indeterminate (o–o edge).

## 4 Discussion

This study used a data-driven causal discovery approach to examine the directionality of relationships among symptoms, cognition, and real-life functioning in schizophrenia. The resulting Partial Ancestral Graph (PAG) revealed a coherent internal structure, with a central neurocognitive–metacognitive–functional system of directed connections among variables and two additional, partly independent contributions to real-life functioning: conceptual disorganization (Dis) and experiential negative symptoms (Avl). Several other domains, namely positive (Pos) and depressive symptoms (Dep), social cognition (SC), and clinical insight (UoS and MoS), emerged as peripheral, consistent with prior evidence from network-based and longitudinal models of the illness.

Within the main network, visual learning (VisL) emerged as the initial node projecting toward attention/vigilance (Att) and working memory (WM). The latter, together with verbal learning (VerL), projected onto processing speed (SoP), ultimately leading to reasoning and problem-solving (R&PS) ability. This defined a “hierarchical” architecture of executive performance within the neurocognitive network, consistent with evidence showing robust associations of processing speed/attention and working memory with higher-order reasoning and problem solving (22, 23, 76–78).

One of the most notable findings of this study is that working memory (WM) serves as a true convergence node for cognitive trajectories, mediating between neurocognitive components and metacognition (MC), which, in turn, influences real-life functioning (RLFunc). This configuration aligns with the major literature identifying working memory as a pivotal function of executive control and a fundamental predictor of functional outcomes, and as tightly interconnected with other domains (22, 23, 25, 79). It is also central in connections leading to functioning, as shown both in Giuliani et al. (57)—where it appears as the primary node—and in other major network analyses (6, 48, 76).

In our graph, metacognition (MC) serves as an intermediate hub that translates working memory into real-life outcomes (RLFunc). It plays a role analogous to that of functional capacity in the PAG model by Giuliani et al. (57). These results are compatible with models in which the impact of working memory on functioning operates through “utilization” constructs of cognition (metacognition or functional capacity), with variations depending on available indicators (80). The mediating role of metacognition appears relevant both at illness onset (81) and longitudinally, predicting outcomes independently of symptoms and functional capacity (30). Consistent results also emerge from cross-sectional and network analyses (76). Our analysis thus suggests an additional hierarchical stratification of tasks: to sustain adequate real-life functioning, individuals must not only possess basic neurocognitive abilities and retain and manipulate them through working memory, but also hold the metacognitive awareness necessary to monitor and integrate such processes and mental states—both their own and others’—to effectively address daily challenges and achieve complex real-world goals. The centrality of this neurocognition-to-functioning chain of associations is crucial in schizophrenia, a disorder where cognitive deficits are present even before psychotic onset and remain stable across the illness course, regardless of clinical phase, symptom severity, or pharmacological treatment (33, 34, 82, 83).

Conceptual disorganization (Dis) showed an independent, though potentially mediated, connection with real-life functioning (RLFunc). The literature consistently links disorganization with the neurocognitive domain and executive functions (64, 84), identifies it as a mediator in translating basic cognitive processes into metacognitive constructs (12, 17, 85–87), and positions it—together with negative symptoms and metacognition—as a fundamental determinant of functional outcome, across large associative studies (88), network analyses (6, 24, 48, 76), and the PAG-based model by Giuliani et al. (57). The relative isolation of this psychopathological domain in our analysis supports the hypothesis that disorganization is independent of basic neurocognitive tasks (64, 76, 84).

Experiential negative symptoms (Avl) showed two independent connections, causally influencing both expressive deficits (ExD) and functioning (RLFunc), thus forming an autonomous negative symptom microcircuit. Literature consistently places avolition at the core of the trajectory toward functional impairment, often independently of cognitive deficits (11, 24, 89, 90). Experiential negative symptoms are considered hierarchically more relevant than expressive ones, representing one of the strongest predictors of functional scores (14, 91, 92) and showing widespread and central connections with functioning in major network analyses (6, 24, 48, 76) and with functional capacity in Giuliani et al. (57).

Positive (Pos) and depressive (Dep) symptoms, as well as social cognition (SC), occupied peripheral positions in the PAG, showing no direct causal relationships with the main network.

The marginality of positive symptoms (Pos) aligns with literature documenting weak and heterogeneous associations between psychotic features and cognitive or functional domains (11, 13, 93, 94). The absence of significant links with metacognition may reflect limited symptom variability within our clinically stable cohort. Major network analyses confirm the peripheral position of this domain relative to functioning (6, 24, 48, 76), while in Giuliani et al. (57), they appear downstream of functioning, with disorganization mediating the link.

Similarly, depressive symptoms (Dep) showed no directed connections to cognitive or functional variables. Prior studies indicate that while depression can affect cognitive efficiency during acute or early stages, its influence tends to diminish in chronic schizophrenia, becoming less prominent than negative or disorganization symptoms (94, 95). This attenuation likely explains its peripheral placement in our network, consistent with findings from network analyses (6, 48, 76) and the PAG study by Giuliani et al. (57).

Social cognition (SC) also remained isolated. This may result from limited measurement coverage and the absence of functional capacity in the model—a construct known to mediate the social cognition–functioning relationship in several studies (4, 24, 25, 29)—and found to be central in the Giuliani et al. PAG analysis (57), while other recent network analyses confirm the non-central positioning of this domain (76). The isolation of social cognition in our graph may thus reflect several factors: the exclusive use of the MSCEIT “managing emotions” subscale from the MCCB, which captures only a limited aspect of social cognition; the absence of functional capacity among modeled variables; and the lack of intermediate nodes that, as observed in other networks, mediate the impact of social cognition on functioning.

The two clinical insight dimensions—unawareness of symptoms (UoS) and misattribution of symptoms (MoS)—were connected only to each other via an undirected edge, consistent with a conceptual but nondirectional link. The literature reports inconsistent findings regarding the associations among insight, cognition, mood, and functioning (96–99), while major network analyses and PAG-based models do not specifically include MoS and UoS, limiting comparisons. Our results, therefore, provide exploratory evidence of interdependence between these dimensions but do not clarify their causal hierarchy, warranting further investigation.

The chronic and clinically stable nature of the sample, characterized by mild positive symptoms, likely reduced variability in psychopathology, cognitive deficits, and clinical insight, limiting the detection of directed relationships. Methodologically, the PAGs method lacks temporal information and complete causal criteria (e.g., Bradford Hill). Thus, its findings should be further explored through longitudinal designs and broader assessments, especially of social cognition and functional capacities, using larger samples to account for latent confounders and minimize spurious associations. From a future perspective, we would like to increase the sample size to include these variables, which are of significant clinical and rehabilitative interest. Furthermore, it should be noted that this is a single-center study; therefore, the results obtained can only be partially generalized to other national—and even less so to international—clinical settings.

Beyond these limitations, our model shows a clear internal trajectory that largely overlaps with that reported by Giuliani et al. (57): neurocognitive abilities converge on working memory, which, through metacognition, influences real-life functioning. In parallel, it identifies two autonomous, direct routes—disorganization and avolition—that affect functioning with relative independence from the cognitive chain.

### 4.1 Added value beyond previous PAG models

Compared with previous PAG models, particularly the one proposed by Giuliani et al., the present study incorporates assessments of metacognition and clinical insight. The results suggest that clinical insight is not directly linked to functional outcomes. On the contrary, metacognition appears to serve as a bridge between working memory and real-life functioning, just as was the case for functional abilities in the work of Giuliani et al. (57).

From a therapeutic standpoint, several implications emerge. The central role of working memory underscores the importance of targeted cognitive remediation strategies focused on this domain, even in chronically stabilized patients, especially given documented benefits for functioning (100, 101). A progressive intervention order may be advisable: beginning with visual learning exercises, followed by verbal learning, supported by working memory enhancement, and subsequently modules focused on metacognition and reduction of disorganization. Regarding metacognitive therapeutic targets, several validated approaches exist, such as Metacognitive Reflection and Insight Therapy (MERIT) (102); Metacognitive Training (MCT) (103, 104); and Metacognitive Interpersonal Therapy (MIT) (105, 106). Therapeutic decisions aimed at reducing negative symptoms, especially experiential ones, may also be beneficial.

In conclusion, these directed associative connections can serve as operational guides for clinical practice to improve real-life functioning in individuals with schizophrenia, as they suggest integrated, sequential, and multilevel interventions in which each highly relevant node of the map, namely working memory, metacognition, disorganization, and avolition, represents a potential target for rehabilitation.

## Data Availability

The raw data supporting the conclusions of this article will be made available by the authors, without undue reservation.
